# Photo-Dependent Reflex Seizures—A Scoping Review with Proposal of Classification

**DOI:** 10.3390/jcm11133766

**Published:** 2022-06-29

**Authors:** Jolanta Strzelecka, Dariusz Wojciech Mazurkiewicz, Tymon Skadorwa, Jakub S. Gąsior, Sergiusz Jóźwiak

**Affiliations:** 1 Department of Pediatric Neurology, Medical University of Warsaw, 02-091 Warsaw, Poland; sergiusz.jozwiak@wum.edu.pl; 2 St. Mark’s Place Institute for Mental Health, 57 St. Mark’s Place, New York, NY 10003, USA; dwmazurkiewicz@aol.com; 3 Department of Descriptive and Clinical Anatomy, Medical University of Warsaw, 02-091 Warsaw, Poland; tskadorwa@wum.edu.pl; 4 Department of Pediatric Cardiology and General Pediatrics, Medical University of Warsaw, 02-091 Warsaw, Poland; jgasior@wum.edu.pl

**Keywords:** photic stimulation, seizures, electroencephalography

## Abstract

Children and adolescents are the largest at-risk group for the appearance of reflex seizures or epilepsy syndromes with a photoparoxysmal response. The aim of this study was to present an overview of the literature regarding photo-dependent reflex seizures. Epilepsy with seizures provoked by intermittent light stimulation is a distinct group of epilepsies; therefore, we focused on reflex seizures provoked by different factors whose common feature is the patient’s response to intermittent photic stimulation. A qualitative search of PubMed/MEDLINE, Scopus, EBSCO, and Cochrane Library electronic databases for selected terms was carried out for scientific articles published up to May 2020 outlining the outcomes of control, observational, and case studies. This scoping review was developed and followed in accordance with the Preferred Reporting Items for Systematic Reviews and Meta-Analyses extension for Scoping Reviews. The review of the qualitative evidence for the synthesis of photosensitive epilepsy allowed us to distinguish the following categories: light-induced seizures and light-deprived seizures. Differentiating between intermittent photic stimulation-related epilepsy syndromes and seizures is essential in order to determine the length of appropriate treatment. Photo-dependent reflex seizures make up the majority of this type of disorder among reflex seizures. Since there are many seizures provoking factors in the world around us, it is important to distinguish amongst them in order to be able to protect the patient exposed to this factor. It is recommended that the photostimulation procedure be performed during a routine electroencephalogram study.

## 1. Introduction

Reflex seizures (RS) are a group of epilepsies in which seizures are triggered by a specific stimulus. Some patients report that seizures are sometimes or exclusively caused by general internal factors (such as stress, fatigue, fever, sleep, and menstrual cycle) and external factors (such as excess alcohol, heat, bathing, eating, reading, and flashing lights) [1].

It has been known for over a century that flickering light can trigger seizures in susceptible patients [2]. A lot of information has been obtained about the mechanism of intermittent photic stimulation (IPS) and its role in the symptomatology of seizures in children and adolescents. As a result, IPS has become an almost routine procedure in performing diagnostic electroencephalogram (EEG) recordings with stroboscopic light flashes from 2 to 60 Hz to diagnose epilepsy, or the genetic feature, and to evaluate (non)pharmacological treatment [3,4]. Photoparoxysmal response (PPR) may be responsible for the occurrence of a provoked seizure [1,5,6,7].

During childhood and adolescence, the period when epilepsy most frequently begins (about 75% of cases), the PPR to intermittent light stimulation is most pronounced in EEG [8,9,10,11]. Therefore, children and adolescents constitute the most numerous at-risk group for the occurrence of reflex seizures or epilepsy syndromes with PPR. An actual number of people in the so-called risk of reflex seizures group is unknown. To date, there is no controlled epidemiological data on the frequency of this type of seizure [12]. It is assumed that amongst the 1% of people suffering from epilepsy, 6.5% have reflex seizures. Within the group of reflex epilepsy, light-induced seizures are the most frequently recorded, accounting for 5% of all 6.5% of reflex seizures [13]. The estimated prevalence of suspected epilepsy among Europeans is 5–10%, while in some genetically determined idiopathic epilepsy syndromes (i.e., juvenile myoclonic epilepsy) this number may rise to 90% [14]. This group includes juvenile myoclonic epilepsy (JME), Dravet syndrome, Eyelid myoclonia, and PhS occipital lobe epilepsy. Patients in this group usually require many years of pharmacotherapy, sometimes with unsatisfactory response to treatment. Most of these epilepsies are genetically determined and although PPR is a common trait, their genetic location varies with the type of epilepsy. Mutations in the SCN1A and SCN2A genes have been found in Dravet syndrome. There is considerable gene polymorphism in JME, and the most common location is chromosome 22 EFHC1, GABRA1, GABRD, CACNB4, CASR, Cx-36 in 15q4, GRM4, BRD2 in 6q21 [15,16,17,18]. Mutations associated with juvenile absence epilepsy have been found in the CACNA1H gene. Jeavons syndrome shows remarkable genetic heterogeneity, including mutations in the CHD2 and KCNB1 genes being responsible for this type of epilepsy [19]. The genetic significance in PhS epilepsy was determined, in particular, by focusing on the CHD2 gene after finding it to be the only common gene among the few reported in PhS epilepsy with deletions in the chromosome 15q26.1 region [20].

A mentioned above, even though light-induced epileptic seizures have been documented since the mid-nineteenth century, there are still problems with classifying these types of seizures [7]. The literature on these seizures is limited and the multitude of terms used makes it difficult to classify them unequivocally.

Since the adoption of the International Classifications of Epileptic Seizures (ICES, Commission, 1981) and International Classifications Epilepsy and Epileptic Syndromes (ICE, Commission, 1989), interest in classification and terminology related to epilepsy has remained high [21].

The classifications of seizures and epilepsies published by the International League Against Epilepsy (ILAE) in 1981 and 1989, respectively, were intended to increase the precision of the classification and consider newly identified syndromes and their etiology. The 1989 ILAE classification included both specific electroclinical syndromes and broad syndromes based on seizure types and etiology. It was then proposed to distinguish a group of reflex epilepsy, which, in 2001, was included in the syndromes of reflex epilepsy [22,23]. It allowed for the distinguishment of a group of patients exposed to seizures provoked by external stimuli. Reflex epilepsies had been divided into two groups. The first group included those caused by simple sensory stimulation—auditory and visual somatosensory stimulation. The second group are those induced by complex stimulation such as: reading epilepsy, musical epilepsy, eating epilepsy, thinking epilepsy [3,24]. Kasteleijn, in 2001, proposed a terminology and classification of clinical and neurophysiological phenomena relating to visual sensitivity. It aims to standardize the use of clinical terms and definitions. This proposal is divided into four main areas: Clinical manifestations of visual sensitivity, Classification of EEG responses to IPS, Classification of electroclinical phenomena, and Classification of syndromes [25].

ILAE 2017 classification modified the division of epilepsies and classified them into three levels, first by seizure type (focal, generalized or unknown), then by type of onset (focal, generalized, or unknown), and then by specific epilepsy syndromes [26,27]. Although the etiology is considered at every stage, broadly defined epilepsy syndromes are not considered. Inappropriate diagnosis of isolation reflex seizures can be a problem in the diagnosis and prognosis of this group of patients. Since epilepsy with PPR affects a large group of patients, it is worth distinguishing these groups of patients in order to properly qualify them for long-term anti-epileptic treatment.

The latest ILAE 2022 classification updated diagnostic criteria for epilepsy syndromes of different ages of onset are presented. These criteria are in line with the currently accepted classifications of epilepsy and seizures, and knowledge from advances in genetics, electroencephalography and imaging is being used. Some reflex epilepsy and PPR epilepsy are distinguished [28].

Due to the multitude of terms and definitions used in the literature, it seems important to focus on photo-dependent reflex seizures and carry out their proper differentiation [14].

The aim of this study was to review the literature to present and compare the type of light stimulants provoking photo-dependent reflex seizures and possible epileptic discharges on the EEG. Secondary aim was to propose a classification of types of photogenic seizures.

## 2. Materials and Methods

### 2.1. Search Strategy

A qualitative research of the PubMed/MEDLINE, EBSCO, Scopus and Cochrane Library electronic databases for the terms “epilepsy” AND “photoparoxysmal response” OR “reflex seizure” OR “television-induced” OR “video-game induced” OR “pattern-induced” OR “self-induced”; and “epilepsy” AND “fixation-off” OR “scotosensitive” was conducted for relevant critical articles in May, 2021, outlining the outcomes of control studies, case studies, and observational studies regarding the main topic, which were then evaluated.

### 2.2. Methodological Quality

This scoping review was conducted and reported according to the Preferred Reporting Items for Systematic Reviews and Meta-Analyses (PRISMA) extension for Scoping Reviews (PRISMA-ScR) [29].

### 2.3. Eligibility Criteria

We implemented the following PICOS criteria:

P (patients)—pediatric participants with photo-dependent reflex seizures;
I (intervention)—all types of stimulation: self-, pattern-, TV-, video-game induced and fixation-off, scotosensitive induced;
C (comparison)—studies comparing different stimulants;
O (outcomes/results)—effect of stimulation, i.e., detection of reflex seizures and/or epileptiform discharges in EEG;
S (study design)—full-text original research, regardless of the study type.

We included full-text journal articles including pediatric patients with reflex photo-dependent seizures. Only studies published in English were included. Articles that did not fulfill the inclusion criteria were excluded from the analysis. Non-full-text articles (e.g., conference abstracts) were not included.

### 2.4. Data Collection Process

JS and DWM independently reviewed the search results to identify any articles that fulfilled the inclusion criteria. Differences between the authors were resolved by discussion and consensus. We juxtaposed and compared authors’ names and patient characteristics. The following information was extracted from each included article: title, authors names, year of publication, sample size and participants characteristics, type of intervention, and results.

### 2.5. Summary Measures

The relationship between stimulating factors and the occurrence of a seizure was searched in included studies. Due to the variety of assessed stimulating factors, we specified the type of stimulation in each study. In addition to distinguishing the stimulating factors, the characteristic features most frequently stimulating and responsible for the onset of an epileptic seizure were selected in each one.

## 3. Results

### 3.1. Study Selection and Available Literature

The search of PubMed/MEDLINE, EBSCO, Scopus, and Cochrane Library provided a total of 390 articles. After adjusting for duplicates, 110 studies remained. Publications in which title and abstract review did not meet the research criterion or publication not linked to the research topic were removed.

**Figure 1:**
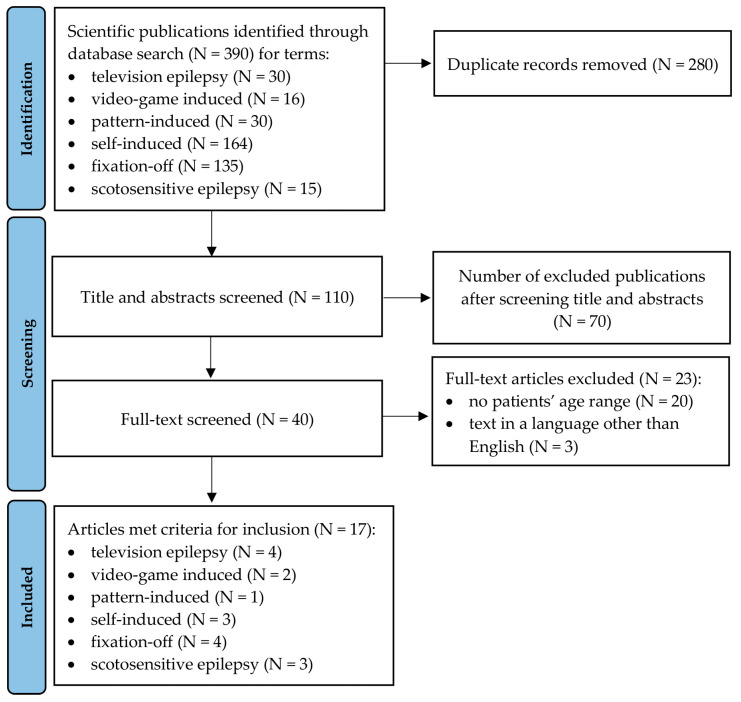
Flow diagram of the screening process.

Of these, 70 reports were excluded in the first and second phase of the screening as they did not meet inclusion criteria or met exclusion criteria (articles not focusing on PDRS). Another 23 reports were excluded in the third phase of the search due to the reasons included in the flow diagram (Figure 1). Studies presented results for sample with a broad age range including adults without separate data presentation for only pediatric participants were included. Overall, 17 reports were included in the review [5,30,31,32,33,34,35,36,37,38,39,40,41,42,43,44,45] with three of the included articles being case reports. Based on the guidelines, 17 publications linked to the research topic met the criteria for inclusion in the scoping review (Figure 1).

### 3.2. Study Characteristics

Patients: patients aged 5–18 years with PDRS.

Intervention: 6 types of stimulators were found: self-, pattern-, TV-, video-game induced and fixation-off, scotosensitive induced. The following types of interventions were applied: monochrome and colour TV, small and large TV screen, visual stimulation of low luminance, deep red flicker and flickering geometric pattern, different types of video games, different patterns: window screens, garments, tablecloths, ceiling tiles, openeyes/close eyes, and darkness.

Primary Outcome: seizure types: absence, myoclonic, partial seizures, GTCS, EM, seizure consisted of visual auras (subjective symptoms), tonic and versive seizures, and autonomic seizures. EEG recordings revealed generalized epileptiform discharges, focal epileptiform abnormalities, abnormal background activity, PPR, spike-and-wave discharges evoked by eye closure and by darkness, and blocked by eye opening.

Study design: No studies were randomized. Four of them were retrospective and five studies were prospective. One article included a cross-sectional study. Three studies were case series or case studies. These cases involved the very rare PDRS-scotosensitive epilepsy.

### 3.3. Qualitative and Quantitative Synthesis Findings

The findings of the qualitative synthesis data collection of 17 researched publications are demonstrated in Table 1.

### 3.4. Overall Assessment of Patients and Kinds of Stimulation

Articles selected for review included groups with different numbers of patients. Studies that presented photic stimulation included a larger number of patient than studies without photic stimulation. Several studies included patients being treated for epilepsy, and these groups were comprised of selected patients with reflex seizures only. This study found that most of the reflex seizures were provoked by video games. A video game study looked at the type of game and found Super Mario World to be the most provocative game. The study on TV induced seizures evaluated the effects of large and small screens. Another study compared monochrome and color television. One article evaluated the effects of low and high luminance and wavelength on seizure onset. Two articles with multicenter studies were found in the literature, one on seizures provoked by video games and the other on patients with fixation-off epilepsy. The study with video games included 352 patients. The second study involved a group of 200 patients with fixation-off seizures.

In a study with self-induced seizures, fenfluramine, which is not a well-performing antiepileptic drug, was used for treatment. Seven patients became seizure free and others had a reduction in seizure frequency of more than 75%. Case reports were found for light-deprived seizures, but no found article contained a bigger group of patients despite numerous reviews. Thus, it can be argued that these seizures can be difficult to diagnose.

### 3.5. Proposal of Photodependent Seizures Classification

- Reflex epilepsy syndromes

Reflex epilepsy syndromes is a broad term referring to various types of epilepsy and epilepsy syndromes in which, in addition to spontaneous seizures, there are seizures provoked by photostimulation. In EEG, localized or generalized discharges occur in the basic examination and during photostimulation.

- Photo-dependent reflex seizures (PDRS)

Photo-dependent reflex seizures only occur during IPS, i.e., visual (external) stimuli. Spontaneous seizures are not observed. A basic EEG test performed in most patients showed no abnormalities. Seizure disorders are recorded only during IPS in order to detect abnormal epileptogenic sensitivity to flickering light in diagnostic EEG recordings. The response to PhS is the major abnormality in the EEG.

**Figure 2:**
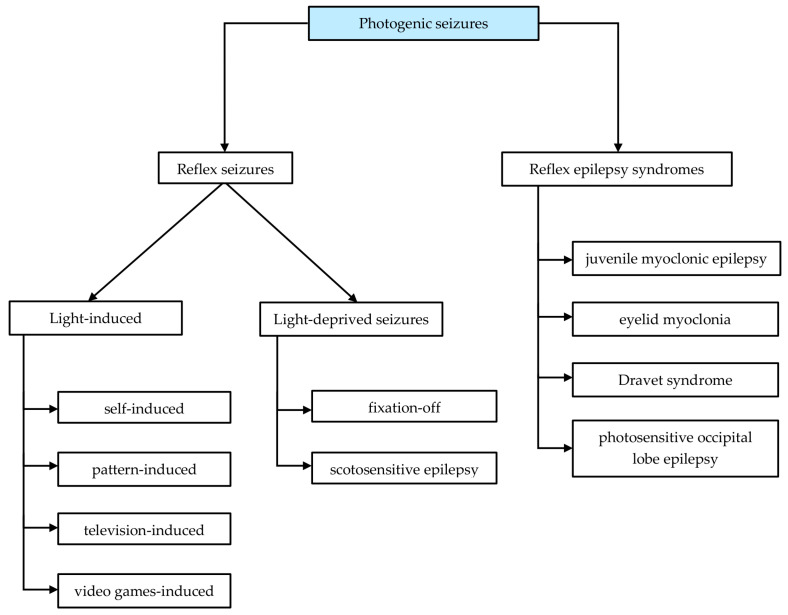
Classification of photodependent seizures.

A review of the literature along with authors’ experiences allow to propose a classification of types of photogenic seizures (Figure 2):

## 4. Discussion

Stimulation with a photic signal (TV, pattern, VG, self-inducted) or elimination of central vision (FOS, scotosensitivity) induced PDRS in patients aged 5–18 susceptible to light stimuli.

We classified PDRS into the following categories: light-induced seizures and light-deprived seizures. We distinguished between self-induced, pattern-induced, television-induced and videogame induced in light-induced seizures. We distinguished between fixation-off and scotosensitivity seizures in light-deprived seizures (see Figure 2).

Patients with epilepsy and PPR can have generalized and focal seizures [42,46,47]. Although generalized seizures, such as myoclonic, tonic-clonic, and absence, are the most common, PPR has also been found in patients with occipital epilepsy and temporal lobe epilepsy [47]. According to the study by Yang Lu (2008), PPR was observed in 20% of patients with focal seizures [48]. Focal seizures are recorded as visual, motor, and sensory disturbances. In photogenic epilepsy, both types of seizures can occur in the same patient. IPS is the leading symptom in photogenic epilepsy. This group includes the so-called pure reflex epilepsy, in which the seizures are caused by different types of light—IPS, patterns, computer games—or by a lack of light. When a seizure trigger is identified, the patient usually avoids the situations or factors that provoke the seizure.

Radovici first described eyelid myoclonia and absence seizures in response to eyelid closure while looking at bright lights [49]. The occurrence of seizures is related to the phenomenon of autophotostimulation [38]. Patients feel positive emotions and relaxation during the seizure, thus leading to self-induced seizures [50,51]. Since patients provoke a seizure by stereotypical hand movements, this epilepsy can be confused with tics. Patients stare at a light source and wave abducted fingers in front of their faces while slowly closing their eyes or perform other behaviors that create a similar flicker effect. This constellation of symptoms has been termed “Sunflower Syndrome” due to the sun-seeking behaviors of the patients and the characteristic way in which they bend their faces up toward the sun [36]. Some patients have seizures that are sensitive to certain patterns. The most common are patterned clothes (grids, stripes), window blinds, escalators, and ceiling tiles [35]. Although most pattern-sensitive patients are also PhS, pattern sensitivity may occur in isolation. Pattern-provoked epileptic activity arises in the visual cortex. However, it has been noted that the topography of EEG activity in response to patterns is usually mainly located over the posterior temporal and parietal scalp electrodes rather than the occipital electrodes [12].

In the 20th century, with the development of electronic media and the increasing spread of TV, it was noticed that some people experience epileptic seizures while watching TV. The first study of epilepsy and PhS patients (n = 454) was by Jeavons and Harding in 1975 [52]. It found that 35% of patients had seizures only while watching TV, without spontaneous seizures [10]. In 1979, based on the conducted research, Wilkins et al. found that the percentage of PhS patients affected by television increased dramatically when the viewing distance was reduced. Screen size matters [30]. A television with a small screen, stimulates a smaller area of the retina than that with a large screen. The results of the study indicated that this increase in sensitivity is due to two factors; firstly, an increase in the amount of stimulated retina and secondly, an increasing resolution of the lines that make up the image. Etemadifar et al., in a TV epilepsy study, found that girls suffered more than boys. In this study, 43.3% of the patients had co-epileptic syndromes, all of them with generalized idiopathic epilepsy, and 56.7% of the patients had pure TV epilepsy [32].

In December 1997, after a four-second animation of a blue-and-red Pokémon was broadcast in Japan, 687 people from all over the country, mostly children, were hospitalized due to seizures [31]. For 76%, it was the first seizure in their lives. During the Olympics Games in London in 2012, the organizers had to change the TV logo of the event due to the appearance of epileptic seizures in viewers.

In some patients, seizures due to hypersensitivity to flashing lights occurred only while playing computer games. Epilepsy associated with playing video games (VG) has been recognized since the early 1980s, with the first definitions being “Space Invader epilepsy”, “Dark Warrior epilepsy”, and “Nintendo epilepsy”. VG is a combination of visual stimuli (screen flickering, program color, patterns and brightness, and the impact of the screen scanning) and other factors such a practice and mental activity. The frequency of seizures provoked by the current widespread nature of video games is not precisely defined. This disease most often affects people aged 9–19 years, more often boys, who spend a significant part of their free time playing computer games. Seizures due to video games have been reported in patients with PhS and non-photosensitive epilepsy. Patterns including epileptiform activity with a maximum concentration over the posterior temporal and parietal scalp, rather than the occipital region as is typical in IPS have been shown [53]. It is, therefore, likely that VGs containing multiple patterned images will trigger a different type of seizure when closely examined. Some games are more provocative than others depending on the brightness levels and combinations of colors and blinking lights. According to a study by Kasteleijn-Nolst et al., Super Mario World triggered the first seizure in many patients and proved to be more epileptogenic than standard TV shows and as provocative as shows with flashing lights and patterns [5,34].

Seven articles present seizures provoked by lack of light or lack of fixation of eyesight. There are two types of stimulation—fixation-off and scotosensitivity [39,40,41,42,43,44,45].

Although the majority of people with visually provoked seizures and vision sensitive epilepsy are PhS, pattern-sensitive, or both, there have been a few reported patients with seizures that occur when their eyes are closed, or their focal point vision deteriorates.

The term fixation sensitivity (FOS) was originally used by Panayiotopoulos for epilepsy, or EEG abnormalities, or both, caused by a lack of central vision and fixation [54]. FOS can occur in both nonphotosensitive and PhS patients and can be in mild and medically intractable epilepsies, as well as in patients without obvious epileptic seizures [39].

Another type of stimulation is scotosensitivity, which is defined as epileptic discharges induced in pure darkness. This is a very rare type of epilepsy. We found three articles on this topic, each one describing individual patients. PhS epilepsy, scotosensitive epilepsy and FOS are often revealed in a resting EEG. Therefore, it is important to distinguish between eye-closure and eye-closed EEG abnormalities due to their different properties and their different responses to intermittent light, darkness and fixation. Eye-closure induces mainly generalized seizure activity that occurs within 2–4 s after closing the eyes, usually lasting 1–4 s [42].

In the assessment of general characteristics of the patients, such as sex and age, we have no observed important differences in answer for IPS, but we would like to emphasize that males are more sensitive to VG while females are more sensitivity for TV induced. It may be connected to the higher frequency VG use by males than females.

### Limitation of the Study

Some articles could be omitted in the literature search, given that the search terms and language restriction used.

## 5. Conclusions

PDRS are the most common of this type of disorder among reflex seizures. This study summarizes the current knowledge about the risk factors related to an increased risk of PDRS in individuals with PPR. Since there are many seizure provoking factors in the world around us, it is important to distinguish amongst them in order to be able to protect the patient exposed to this factor. Photostimulation should always be performed during routine EEG testing, even if the patient does not report any symptoms. The purpose of PS is to identify patients with PPR and determine the appropriate treatment to avoid photostimulation or, if necessary, introduce early treatment to avoid seizures.

## Figures and Tables

**Table 1:** Qualitative synthesis findings.

**Author, Year**	**Patients**	**Intervention**	**Comparison**	**Outcome**	**Study Design**
**Light-induced seizures**					
***Television-induced***					
Wilkins A. et al. (1979) [30]	N = 21: F:M = 4:3; age 6–31 years	TV induced - patterned and diffuse IPS, - monochrome and colour television, - static and moving patterns of stripes	Lack of comparisons	From 21 patients: - 16 patients were sensitive to diffused IPS, - 19 patients were sensitive to patterned IPS, - 16 patients were sensitive to the monochrome set, - 13 were sensitive to the colour TV	prospective study non-randomized
	N = 8: M = 6, F = 2; age 11–42 years	TV induced - small TV screen, - large TV screen, - large TV screen covered by a mask containing a central aperture the same size as the small screen	- large screen—distance 3 m - small and masked screen—distance 1.5 m	Proportion of PhS patients affected by television increases as the viewing distance is reduced; small screen television was less epileptogenic than a large screen television viewed at the same distance	prospective study non-randomized
Takahashi T. et al. (1998) [31]	N = nearly 700 people, mostly children	TV induced - Pokemon Monsters program - visual stimulation of low luminance - deep red flicker and flickering geometric pattern stimuli with 10–20 cdm^2^ luminosity - ordinary high luminance stroboscopic IPS	Lack of comparisons	Healthy youngsters may have latent PhS and sensitivity might be disclosed by use of low luminance deep red flicker stimulation. Deep red flicker stimulation is more provocative of PPR than ordinary high luminance stroboscopic IPS	prospective study non-randomized
Etemadifar M. et al. (2008) [32]	N = 1705; N = 30 TV epilepsy: M = 13, F = 17; age < 12 years	TV induced - seizures triggered by watching TV, - abnormal EEG findings, - patients had PPR to IPS	Lack of comparisons	57% pure TV epilepsy: patients had: absence (3.3%), myoclonic (3.3%), GTCS (93,3%) seizures in response to IPS; 43% TV epilepsy and other types generalized seizure	retrospective study non-randomized
Brinciotti M. et al. (2015) [33]	N = 26: M = 12, F = 14; mean age 14 years	TV induced Video-EEG recordings: - at rest - during IPS, - during pattern stimulation (PS), - during TV watching Blink rate was evaluated: - at rest, - during a TV-viewing period, - during the occurrence of PEM	Lack of comparisons	EM were recorded in all patients. The frequency of EM ranged from 8 to 12.5 Hz (average: 9.6 ± 1.5). Visually-induced seizures were recorded in 20 patients, triggered by stimuli (IPS and PS) in 11 patients; seizures were triggered by PS (but not IPS)—5 patients, IPS (but not PS)—3 patients, TV watching (but not PS or IPS)—1 patient	prospective study non-randomized
***Video-game induced (VG induced)***					
Piccioli M. et al. (2005) [34]	N = 29: M = 12, F = 17; mean age 14.7 years there were selected those with generalized epileptiform discharges of at least 0.5-s duration during VG provocation: 8 M, 7 F;	VG induced Children were visually stimulated with IPS and black-and-white striped patterns before they started playing the VG (Super Mario World, Super Mario, Mario Kart, Street Fighter II, Super Bomberman II, The Magical Quest, Super Mario All Stars, Super Aleste)	Comparison symptoms evoked by VG and IPS	All patients showed generalized epileptiform activity either spontaneously or evoked by IPS and by playing VG (all). The majority reacted to 4–5 of the 12 VG tested, while Super Mario World was provocative in all patients.	retrospective study non-randomized
Kasteleijn-Nolst Trenite D.G.A. et al. (2002) [5]	N = 352: M = 41% F = 59%; age 13–18 years	VG induced Different types of video games	Lack of comparisons	83% (N = 294) had a history of epileptic seizures, visually-induced seizures were in 77% (N = 225) of those with a seizure history. VG Super Mario World was the most provocative	prospective study non-randomized
***Pattern-induced***					
Radhakrishnan K. et al. (2005) [35]	N = 73; M = 30, F = 43; median age 12.8 years	Pattern-induced Different patterns: window screens, garments, tablecloths, and ceiling tiles	Lack of comparisons	Patients exhibited absence, myoclonic, partial seizures, GTCS in various combinations	retrospective study non-randomized
***Self-induced***					
Baumer F.M. et al. (2018) [36]	N = 13: F = 77%, M = 23%; age 2–8 years	Self-induced—Sunflower Syndrome The background in EEG was normal. 10 patients had generalized 3–4 Hz spike wave discharges, with a bifrontal predominance; 9 of the 10 had multiple runs lasting longer than 3 s. 8 patients had polyspikes or frontal fast activity	Lack of comparisons	5 patients had EM 6 patients—EM and absence 2 patients—absence seizures	retrospective study non-randomized
Barnett J.R. et al. (2020) [37]	N = 24: F = 18, M = 6; age 6.4–25 years	Self-induced—Sunflower Syndrome	Lack of comparisons	Sunflower syndrome—generalized, pharmacoresistant epilepsy with childhood onset Absence seizures	retrospective study non-randomized
Boel M. et al. (1996) [38]	N = 11: F = 7, M = 4; mean age 8 years	Self-induced Intellectual disability in all patients.	Lack of comparisons	All patients had GTCS, 5 patients had absence seizures	study reports
**Light-deprivated seizures**					
***Fixation-off (FOS)***					
Koutroumanidis M. et al. (2009) [39]	N = 14: F = 10, F = 4, age 9–48 years	FOS EEG database were analyzed to find all patients with video-EEG-documented FOS	Lack of comparisons	FOS can occur in non-PhS and PhS patients; FOS can be in mild and medically intractable epilepsies, and in patients without obvious epileptic seizures. All had epileptiform activity—focal, generalized or both. An approximate incidence of 0.2%	retrospective study non-randomized
Wang X. et al. (2018) [40]	N = 8: F = 3, M = 5; age 8–14 years	FOS	Lack of comparisons	PhS was in 6 patients. PPR was elicited during IPS at frequencies 10–20 Hz Patients had seizures: 4—EM, 2—JME, 1—PhS epilepsy, 1-GTCS	retrospective study non-randomized
Karkare K.D. et al. (2018) [41]	N = 52: F = 25, M = 27; mean age 10.3 years	FOS Open eyes/close eyes	Lack of comparisons	Seizure consisted of visual auras (subjective symptoms), tonic and versive seizures, autonomic seizures, EM, with or without absences, limb myoclonus and GTCS	cross-sectional study
Dede H.O. et al. (2021) [42]	N = 200: F = 106, M = 94; age 4–80 years	FOS Open eyes/close eyes All the patients encountered with FOS were children in this study	Lack of comparisons	20 of the analyzed EEG recordings revealed generalized epileptiform discharges. 51 recordings indicated focal epileptiform abnormalities, 42 were abnormal background activity, 87 were normal EEG. Seizures: temporal, frontal, occipital, centroparietal, temporo-parieto-occipital	prospective study non-randomized
***Scotosensitive epilepsy***					
Suresh-babu S. (2017) [43]	N = 1: F; 11 years	Scotosensitive epilepsy Open eyes/close eyes	Lack of comparisons	Patient had absence seizures, EM, and rarely GTCS occipital epileptiform discharges which appeared only during eye closure	case report
Agathonikou A. et al. (1998) [44]	N = 1: M; 16 years	Scotosensitive epilepsy Open eyes/close eyes Central vision and fixation were eliminated with vision through +10 spherical lenses or underwater goggles covered with semitransparent tape	Lack of comparisons	IPS elicits generalized discharges of multiple spike and slow waves even when the eyes are open, and the ictal clinical manifestations enhance when IPS is combined with eye-closure, absence and myoclonic jerks	case report
Lugaresi E. et al. (1984) [45]	N = 4: F = 1, M = 3; age 12–14 years	Scotosensitive epilepsy Open eyes/close eyes seizures induced by eye closure and darkness	Lack of comparisons	Spike-and-wave discharges evoked by eye closure and by darkness, and blocked by eye opening	cases report

N—number of patients, F—female, M—male, IPS—intermittent photic stimulation, PhS—photosensitivity, PPR—photoparoxysmal response, PS—pattern stimulation, VG—video-game, EM—eyelid myoclonia, FOS—Fixation-off, JME—juvenile myoclonic epilepsy, GTCS—generalized-onset tonic-clonic seizures.

## Data Availability

Not applicable.
